# Implications of component size and calibration error on digital templating for total hip arthroplasty. A visual matrix from a simple mathematical model

**DOI:** 10.1007/s11548-021-02367-4

**Published:** 2021-04-17

**Authors:** Christoph Kolja Boese, Tim Rolvien, Matthias Trost, Michael Frink, Jan Hubert, Frank Timo Beil, Christian Ries

**Affiliations:** 1 Department of Orthopaedic and Trauma Surgery, University Hospital of Cologne, Cologne, Germany; 2 Division of Orthopaedics, Department of Trauma and Orthopaedic Surgery, University Medical Center Hamburg-Eppendorf, Hamburg, Germany; 3 Department of Orthopaedics and Traumatology, St. Josef-Hospital, University of Bochum, Bochum, Germany; 4 Center of Orthopedic and Trauma Surgery, University of Giessen and Marburg, Marburg, Germany

**Keywords:** Joint replacement, Arthroplasty, Hip, Digital templating, Calibration

## Abstract

**Objective:**

Preoperative digital templating is a standard procedure in total hip arthroplasty. Deviations between template size and final implant size may result from inaccurate calibration, templating as well as intraoperative decisions. So far, the explicit effect of calibration errors on templating has not been addressed adequately.

**Materials and Methods:**

A mathematical simulation of calibration errors up to ± 24% was applied to the templating of acetabular cups (38 to 72 mm diameter). The effect of calibration errors on template component size as deviation from optimal size was calculated.

**Results:**

The relationship between calibration error and component size deviation is inverse and linear. Calibration errors have a more pronounced effect on larger component sizes. Calibration errors of 2–6% result in templating errors of up to two component sizes. Common errors of up to 12% may result in templating errors of 3–4 sizes for common implant sizes. A tabular matrix visualizes the effect.

**Conclusion:**

Calibration errors play a significant role in component size selection during digital templating. Orthopedic surgeons should be aware of this effect and try to identify and address this source of error.

## Introduction

Preoperative digital templating is a standard procedure in total hip arthroplasty (THA) to determine the sizes of the definitive prosthetic components [1]. Precise calibration of the digital radiograph is the perquisite for a reliable templating of component size and positioning [2–4]. While a number of calibration methods have been suggested, each of these methods has specific limitations which may result in erroneous calibration [2, 4–6]. The mismatch between preoperative templating and the definitive prosthesis size has been discussed in detail. However, the exact effect of the calibration error on the templating success remains unknown. Previous studies compared preoperative templating with definite intra-operative implant sizes [7]. This approach is simple, but ignores potential intraoperative reasons to deviate from the templated implant size. In other words, these previous studies are based on the underlying assumption of both perfect surgery and faultless preoperative templating. Interestingly, there are no publications that specifically analyzed the effect of calibration errors on templating sizes of components. Templates for prosthetic components are based on computer-aided design (CAD) images. They precisely depict the component without projectional influences in accordance. Therefore, the intercept theorem can be used to plan THA components in digital radiographs with a defined magnification factor [4]. The magnification factor is based on calibration markers or a fixed value [2, 8, 9]. In templating, the relationship between calibration error and component magnification is inverse. Thus, a malpositioned calibration marker to close to the source of the X-ray beam results in an overestimation of the magnified marker and leads to undersized components [3, 4]. This poses a potential risk for an intraoperative deviation from the preoperative templating.

Taking these considerations into account, this study aimed to explain and characterize the effect of the calibration error on digital templating for THA. We specifically aimed to create a precise tabular visual matrix demonstrating the influence of cup size on the success of the templating (i.e., the final cup size).

## Material and methods

A mathematical simulation of the effect of calibration errors on various sizes of acetabular cups was performed. Acetabular cup dimensions between 44 and 66 mm were defined as convenient acetabular component sizes. A large sample of sizes from 38 to 72 mm diameter was added to address any possible size including revision cups. A standard of 2 mm increments was used.

Calibration errors to the extent of ± 12% are common in digital templating and calibration errors of up to 23% were observed in previous studies [6–8, 10–12]. Magnification errors were applied in 1% steps.

Therefore, two datasets (groups) were generated:

1. convenient sample of common errors (± 12%) and implant sizes (44 to 66 mm).
2. large sample of possible errors (± 24%) and possible implant sizes (38 to 72 mm).

The magnification error and the true acetabular component size (manufacturer information) were consequently known variables in this model. The projected size of the acetabular shell was calculated considering the magnification error. The formula to calculate the magnified size of the acetabular component is:

$$ {\text{True}}\;{\text{component}}\;{\text{size}}\;{\text{in}}\;{\text{mm }}* \, \left( {100 + {\text{magnification}}\;{\text{error}}} \right)/100 $$

The relationship between calibration error and component size projection is linear.

A matrix was generated for the range of errors and component sizes. The differences between projected and true component size were calculated. It was assumed, that the calculated acetabular component size which was closest to the true component size would have been chosen for preoperative templating. A color-coded table was generated to visualize the expected component selection error in relation to true (optimal) component size and magnification error. Errors above eight sizes were combined into one group. The calculations were visualized for dataset 1 (convenient sample, group 1) and dataset 2 (large sample, group 2).

## Results

**Fig. 1:**
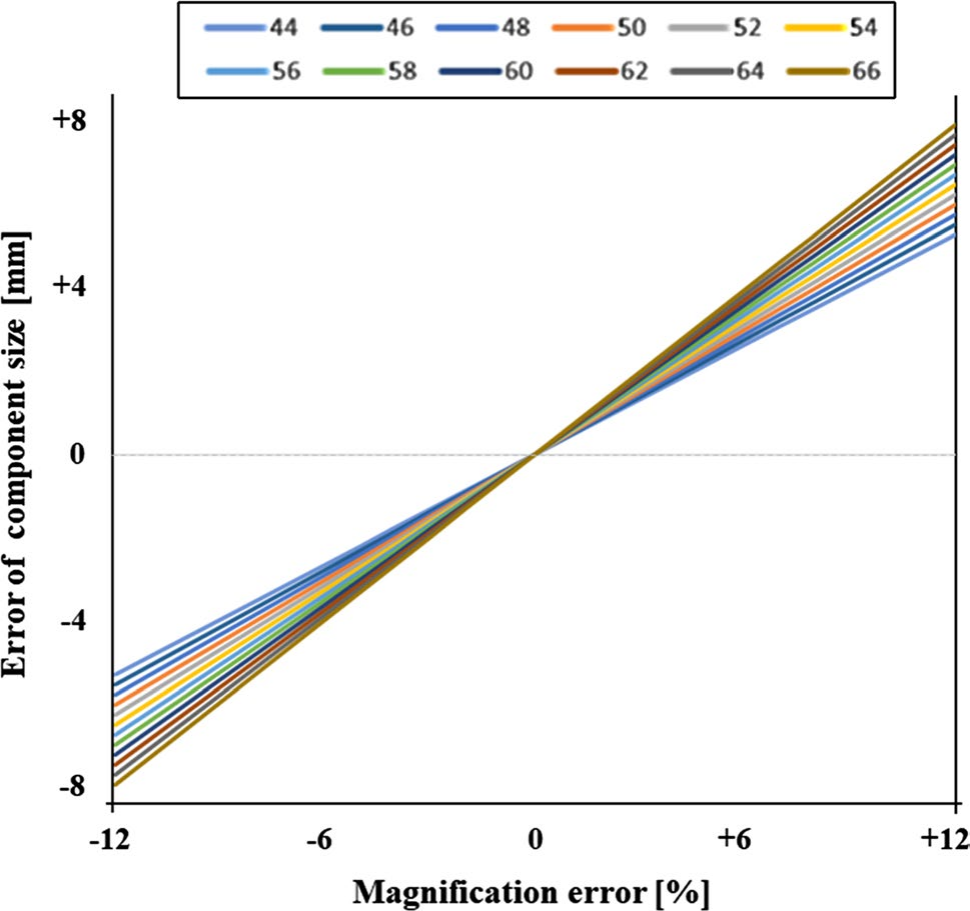
Group 1: Convenient sample. Effect of calibration error (*x*-axis) in percent on the projected component size (*y*-axis) in mm. Lines show component sizes of 44–66 mm

The linear relationship between the magnification error (in percent) and the error of component size (in mm) for each component is visualized for the convenient sample (group 1, Fig. 1). The effect of the magnification error increases with component size. From this linear association, a color-coded table to visualize the expected component selection error in relation to component size and magnification error was created (Table 1).

**Table 1:**
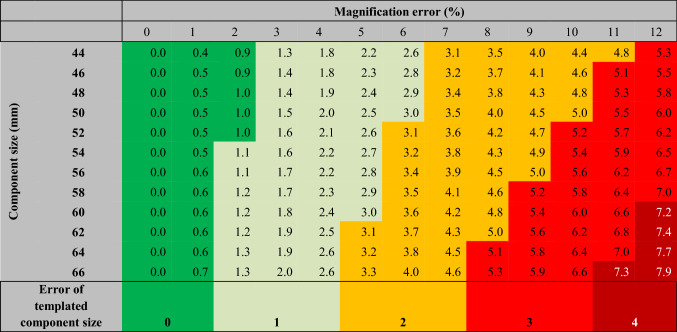
Group 1: Convenient sample. Color-coded table to visualize the expected component selection error in relation to component size and magnification error. Error in component size increases with size of selected component. Lines show component sizes of 44–66 mm. Due to symmetry, only right (positive) values were reported. The values in the table (one decimal) show the planned cup size deviation from the optimal size in mm (component size error)

*Example* Two ways are possible. Starting with a calibration error of 8%, a templated cup of 56 mm diameter should have been a 52 mm cup instead. Thus, an error of two component sizes (or 4 mm) resulted. Alternatively, starting with the optimal cup size of 52 mm, the matrix shows the resulting error per calibration error. In the example of 8%, two sizes difference would have resulted (i.e., 56 mm).

**Fig. 2:**
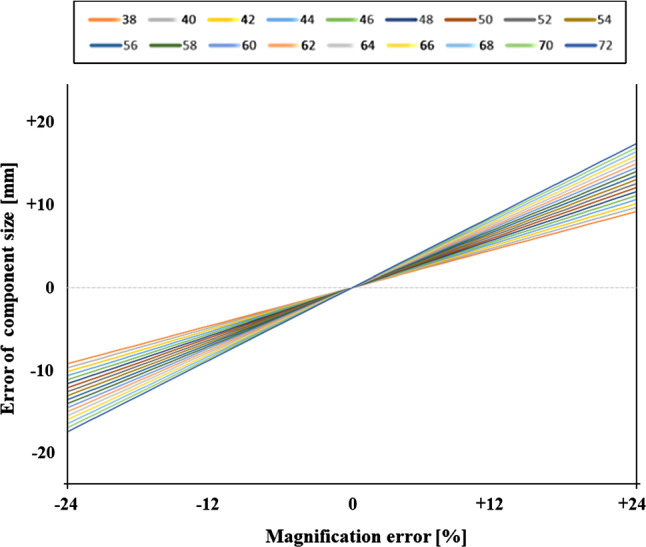
Group 2: Large sample. Effect of calibration error (x-axis) in percent on the projected component size (y-axis) in mm. Lines show component sizes of 38–72 mm

We additionally applied the model to the large sample (group 2), where the similar linear relationships are applicable (Fig. 2). The visual matrix shows that larger acetabular cups have a larger effect on the error of component size (Table 2).

**Table 2:**
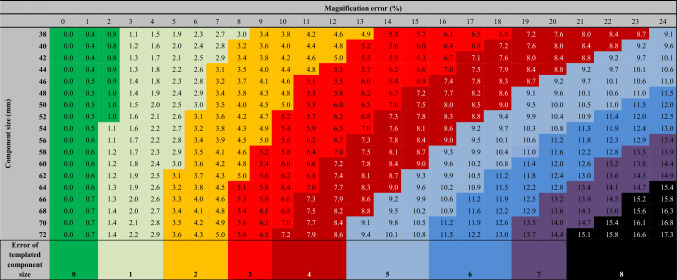
Group 2: Large sample. Color-coded table to visualize the expected component selection error in relation to component size and magnification error. Lines show component sizes of 38–72 mm. Due to symmetry, only right (positive) values were reported. The values in the table (one decimal) show the planned cup size deviation from the optimal size in mm (component size error)

For the chosen range of errors and component sizes, the templating error reaches up ± 7.9 mm equivalent to approximately ± 4 component sizes in group 1 and up to ± 21.1 mm equivalent to approximately ± 10 component sizes in group 2. Color-coded Tables 1 and 2 show the distribution of expected differences between final and templated component sizes providing a detailed presentation of the data.

## Discussion

Radiographs of the hip or pelvis are subject to magnification effects which need to be considered during templating [4]. Therefore, calibration markers are used to indicate the magnification of the targeted plane of the hip. Mostly, radio-opaque objects (e.g., markerball) are used [2]. While distinct projectional characteristics of spherical needed for calibration of radiographs require more complex mathematics, most software and manual methods to calculate the magnification factor follow the simplified intercept theorem [4]. In digital templating, the magnification or calibration factor is applied to the template of prosthetic components. Thus, they can be placed and fitted to the radiograph for preoperative planning. The positive benefit of preoperative templating in total joint replacement is generally accepted and an integral part of quality management.

This study aimed to explain and characterize the specific effect of the calibration errors on digital templating for THA. We showed that the calibration error directly influences the selected implant size in an inverse and linear manner. Notably, the error of selected implant sizes increased with size of the optimal implant size. For the most common implant sizes in combination with likely calibration errors of one or two component sizes are to be expected frequently. However, even errors of three—and for large components four sizes—are not unlikely given the published experience on calibration errors.[6–8, 10–14]

There are several studies comparing preoperative templating with implanted component sizes [6, 7]. However, these comparisons combine various aspects of pre- and intraoperative procedures. While calibration errors might play a role, the individual interpretation of the planner, as well as intra-operative decisions, influences the difference between definitive implant size and preoperative template. Until now, there is no mathematical method to predict the effect of the calibration error on the template. The present study is the first approach to analyze and explain this effect in detail. For reasons of comparability, only acetabular components were considered. Most manufacturers provide hemispherical cups with 2 mm increments in size. Thus, the assumptions can easily be transferred to other acetabular components.

A mathematical calculation of the expected magnification of acetabular components in dependence of the true component size and the calibration error was performed. A simplified categorization into expected component size-differences was performed. While the convenient sample of cups ranging from 44 to 66 mm is of highest clinical significance, the sample using larger implants describes the trend seen with growing templated components. Generally, we demonstrated that the effect of the calibration error is more pronounced the larger a component is planned.

A simplified color-coded table was presented to quickly identify the effect of calibration error on component size. It might help to decide whether a suspected calibration error might result in a relevant templating error. In particular, our model creates awareness and the ability to estimate the error of the templates. However, when the calibration of a radiograph is of doubtful quality, two common options are available. On one hand, the radiograph can be repeated, and the calibration marker position optimized. This has two disadvantages: higher radiation exposure and possibly repeated malpositioning of the marker. On the other hand, a fixed magnification factor could be applied. Sinclair, Franken and Boese et al. independently found a higher precision of the calibration using fixed factors [2, 6, 8]. Alternatively, a dual marker calibration could be used [8].

In conclusion, this study showed the high relevance of correct calibration before digital templating in THA. Although very simple, our mathematical model and the associated visualization have demonstrated for the first time how, assuming a certain magnification error, a larger implant leads to a larger error in component size. Surgeons should be aware of the effect, that larger implants increase the error of implant size for the same calibration error compared to smaller components. The combination with methods to identify misplaced calibration markers might improve patient safety. In the future, templating software should provide an estimated range of the calibration and sizing error to improve the preoperative assessment in THA.
